# Rapid Increase in Estimated Number of Persons with Atrial Fibrillation in Japan: An Analysis from National Surveys on Cardiovascular Diseases in 1980, 1990 and 2000

**DOI:** 10.2188/jea.15.194

**Published:** 2005-09-27

**Authors:** Masaki Ohsawa, Akira Okayama, Kiyomi Sakata, Karen Kato, Kazuyoshi Itai, Toshiyuki Onoda, Hirotsugu Ueshima

**Affiliations:** 1 Department of Hygiene and Preventive Medicine, School of Medicine, Iwate Medical University.; 2 Department of Preventive Cardiology, National Cardiovascular Center.; 3 Department of Health Science, Shiga University of Medical Science.

**Keywords:** the National Survey of Cardiovascular Diseases, Atrial Fibrillation, Prevalence, Japan, general population, projected population

Atrial fibrillation (AF) is one of the common arrhythmia and a potent risk factor associated with increased cardiovascular morbidity and mortality. Not only mortality but also burden of disability and medical cost associated with AF are critical issues. Prevalence of atrial fibrillation increases with advance of age. The number of adults with AF has been increasing with the aging population in western countries.^1^

AF is also an important disease in Japan. Ischemic strokes associated with cardiogenic embolism accounted for 20% in Japanese general population, and an approximately three fold excess risk of cerebral infarction was observed in subjects with AF.^2^ The prevalence and incidence of stroke in Japan are higher than those in western countries. Japan has the most rapidly aging population in the world. It is important to determine the trends in the prevalence of AF and to estimate the numbers of Japanese persons with AF currently and in the future. However, there is no report in which trends in the prevalence of AF in Japan are described, and there are only a few reports in which the prevalence of AF among Japanese is mentioned.

In this study we compared the sex- and age-specific prevalences of AF (proportions of persons with AF in each age- and sex-specific group) in the Japanese general population over the past two decades. We also estimated the number of Japanese with AF in the past and predicted the number of Japanese adults with AF in the future.

## SUBJECTS, METHODS, AND RESULTS

A national survey on cardiovascular diseases in Japan has been carried out every 10 years. About 300 districts were selected using a stratified random sampling method based on the latest national census. All residents aged 30 years or older were invited to take part in this survey. A total of 27,121 persons (10,897 in 1980, 8,926 in 1990 and 7,298 in 2000; response rate: 78.9%) participated in three national surveys.^3^^-^^5^ Resting 12-lead electrocardiograms were recorded in 23,713 participants (10,376 subjects (75.0%) in 1980, 8,139 subjects (74.3%) in 1990, and 5,198 subjects (62.2%) in 2000). We used data from 23,713 participants (10,042 men and 13,671 women). This study is based on published reports from three national surveys.^3^^-^^5^

The electrocardiographic findings were independently evaluated by two trained physicians according to the Minnesota Code. In cases of inconsistent judgments, the final judgments were undertaken after deliberations of the approval committee. Atrial fibrillation was defined as 8-3 (including paroxysmal atrial fibrillation and atrial flutter) according to the Minnesota Code.^3^^-^^5^

We determined sex- and age-specific prevalences of AF in 1980, 1990, and 2000 (expressed as percentages). The averaged sex- and age-specific prevalences of AF were calculated using pooled data of three surveys. The averaged sex- and age-specific prevalences were applied to the Japanese census data (1980, 1990, and 2000) to calculate the number of adults with AF. Furthermore, the averaged sex- and age-specific prevalences were applied to the projected Japanese census data (using medium variant estimates in 2010, 2020, and 2030) provided by National Institute of Population and Social Research^6^ to calculate the number of Japanese adults with AF in the future. The prevalences of AF in groups were compared using the chi squared test. A p value less than 0.05 was considered to be statistically significant.

Table 1 shows sex- and age-specific prevalences of AF for each 10-year age group in 1980, 1990, and 2000. The prevalence of AF in men aged 30 years or older was higher than that in women (1.0% vs. 0.6%; p<0.001). Age-specific prevalence of AF in men aged 70 years or older was higher than that in women (p=0.006), but other age-specific prevalences in each age group (30-, 40-, 50-, and 60-year age group) in men were not statistically different from those in women (p: 0.06-0.17). Prevalence of AF increased with advance of age in both men and women (from 0.1% in adults younger than 50 years to 2.9% in persons aged 70 years or older). The sex- and age specific prevalences of AF in the three surveys were similar.

**Table 1.** Sex- and age-specific prevalence (%) of atrial fibrillation in three national surveys in Japan.

Age (year)	Mean prevalence in 3 surveys	1980	1990	2000	p value
Men					
30-39	0.1	0.2	0.0	0.0	0.46
40-49	0.3	0.3	0.2	0.0	0.55
50-59	0.7	0.8	0.8	0.4	0.70
60-69	1.3	1.0	1.6	1.4	0.64
70+	3.8	3.7	4.3	3.5	0.82
Women					
30-39	0.0	0.0	0.0	0.0	-
40-49	0.1	0.1	0.0	0.2	0.40
50-59	0.4	0.5	0.1	0.7	0.13
60-69	0.9	1.4	0.7	0.5	0.09
70+	2.2	2.6	1.9	2.1	0.67
Both sexes					
30-39	0.0	0.1	0.0	0.0	0.42
40-49	0.1	0.2	0.1	0.1	0.71
50-59	0.5	0.6	0.4	0.6	0.53
60-69	1.1	1.3	1.1	0.9	0.66
70+	2.9	3.1	2.9	2.7	0.84

30+	-	0.7	0.7	0.9	

These data are based on published reports from three national surveys.^3^^-^^5^

Comparisons in the prevalence of atrial fibrillation among three surveys were performed using the chi squared test.

Table 2 shows estimated numbers of Japanese adults with AF in 1980, 1990 ,and 2000. It also shows the projected numbers of Japanese adults with AF in 2010, 2020, and 2030. The estimated numbers of Japanese adults with AF were 391 thousand in 1980 and 729 thousand in 2000. The number of Japanese adults with AF in 2030 is predicted to be 1,081 thousand.

**Table 2.** Estimated numbers of Japanese adults with atrial fibrillation in the past and predicted numbers of Japanese adults with atrial fibrillation in the future (in thousand).

	Calendar year					

	1980	1990	2000	2010	2020	2030
Men	228	306	416	522	598	603
Women	163	229	313	473	457	477
Both sexes	391	534	729	995	1,055	1,081

## DISCUSSION

We found that sex- and age-specific prevalences of AF have not changed in Japan during the past two decades. The prevalence of AF in adults aged 30 years or older has increased from 0.7% to 0.9% with the aging population during this period. The prevalence of AF in men aged 30 years or older was higher than that in women. The prevalence of AF has increased with advance of age in both men and women.

There have been a few reports on the prevalence of AF in Japan. In a hospital-based study, the prevalence of AF among outpatients was 14%.^7^ In a population-based study, it was found that the prevalence of AF in a mixed group of men and women increased from 0.2% in persons aged 40 to 59 years to 2.5% in persons 80 years or older.^8^ The results of our study are similar to those of that study.

The prevalences of AF in adults in western countries have been higher than those in Japan.^1^^,^^9^^,^^10^ The number of adults with AF has been increasing with aging of the population^1^ and the age-specific prevalence of AF has also been increasing in western countries.^9^^,^^10^ It is not clear why the age-specific prevalence of AF has been increasing in western countries. The prevalences of predisposing factors for AF such as past history of myocardial infarction, past history of congestive heart failure, presence of valvular heart disease, obesity, hypertension, and diabetes mellitus have been changing, and these changes might have contributed to the changes in the prevalence of AF in western countries.

In Japan, mean total cholesterol levels and mean body mass index have been increasing in men^5^ and the prevalence of diabetes mellitus has been increasing.^11^ These changes might have elevated the prevalence of AF in Japan. On the other hand, mean systolic blood pressures have been dramatically decreasing in both men and women during the past two decades,^5^ and this decline might have decreased the prevalence of AF in Japan. The different trends in prevalences of predisposing factors to AF in Japan and western countries may explain the different trends in age-specific prevalence of AF in Japan and western countries.

The total number of Japanese adults with AF has almost doubled during the past two decades, and the number will certainly continue to increase with aging of the population. The number of Japanese with AF is expected to exceed one million in 2020. AF is not only a potent risk factor for cardiovascular morbidity and mortality but also a factor that increases both medical costs and burden of nursing care.^12^ We should be aware that an excessive burden associated with AF will certainly impose responsibilities on Japanese people in the future.

There are several limitations of this study. Persons who did not participate in the survey were probably poor health condition and might have had heart disease. Some participants might think that they did not have to undergo electrocardiography because they already had treatment histories of heart disease. These factors might have reduced the number of cases with AF in this study; thus, the prevalences of AF might be underestimated. We applied mean prevalence of pooled data from three surveys to the projected national census because sex- and age-specific prevalences of AF have not changed in Japan during the past two decades. However, the age-specific prevalence of AF will increase in the future if the prevalences of predisposing factors for AF increase as well as western countries, and the number of Japanese adults with AF in the future should exceed the number that we estimated in this study.
